# Establishing and Validating an Aging-Related Prognostic Signature in Osteosarcoma

**DOI:** 10.1155/2023/6245160

**Published:** 2023-02-23

**Authors:** Yibo Ma, Shuo Zheng, Mingjun Xu, Changjian Chen, Hongtao He

**Affiliations:** ^1^Graduate School of Dalian Medical University, Dalian Medical University, Dalian, China 116044; ^2^The Second Ward of Department of Orthopedics, The Second Hospital of Dalian Medical University, Dalian, China 116000; ^3^The Second Hospital of Dalian Medical University, Dalian Medical University, Dalian, China 116000; ^4^The First Ward of Department of Orthopedics, The Second Hospital of Dalian Medical University, Dalian, China 116000; ^5^The Third Ward of Department of Orthopedics, The Second Hospital of Dalian Medical University, Dalian, China 116000

## Abstract

Aging is an inevitable process that biological changes accumulate with time and results in increased susceptibility to different tumors. But currently, aging-related genes (ARGs) in osteosarcoma were not clear. We investigated the potential prognostic role of ARGs and established an ARG-based prognostic signature for osteosarcoma. The transcriptome data and corresponding clinicopathological information of patients with osteosarcoma were obtained from The Cancer Genome Atlas (TCGA) and Gene Expression Omnibus (GEO) databases. Molecular subtypes were generated based on prognosis-related ARGs obtained from univariate Cox analysis. With ARGs, a risk signature was built by univariate, least absolute shrinkage and selection operator (LASSO), and multivariate Cox regression analyses. Differences in clinicopathological features, immune infiltration, immune checkpoints, responsiveness to immunotherapy and chemotherapy, and biological pathways were assessed according to molecular subtypes and the risk signature. Based on risk signature and clinicopathological variables, a nomogram was established and validated. Three molecular subtypes with distinct clinical outcomes were classified based on 36 prognostic ARGs for osteosarcoma. A nine-ARG-based signature in the TCGA cohort, including *BMP8A*, *CORT*, *SLC17A9*, *VEGFA*, *GAL*, *SSX1*, *RASGRP2*, *SDC3*, and *EVI2B*, has been created and developed and could well perform patient stratification into the high- and low-risk groups. There were significant differences in clinicopathological features, immune checkpoints and infiltration, responsiveness to immunotherapy and chemotherapy, cancer stem cell, and biological pathways among the molecular subtypes. The risk signature and metastatic status were identified as independent prognostic factors for osteosarcoma. A nomogram combining ARG-based risk signature and metastatic status was established, showing great prediction accuracy and clinical benefit for osteosarcoma OS. We characterized three ARG-based molecular subtypes with distinct characteristics and built an ARG-based risk signature for osteosarcoma prognosis, which could facilitate prognosis prediction and making personalized treatment in osteosarcoma.

## 1. Introduction

Osteosarcoma is the most common sarcoma mainly occurring in teenagers and young adults around the whole world [1]. It is highly aggressive, and the annual incidence of osteosarcoma is approximately 4.4 per million [2]. Though effective advancement in the treatment and prevention of osteosarcoma has been made over the past decades, overall survival (OS) of osteosarcoma patients is far from satisfactory [3]. Osteosarcoma patients are prone to metastases, and patients with metastatic osteosarcoma have a lower survival rate [4]. More than half of osteosarcoma patients die from metastasis [5]. Meanwhile, osteosarcoma patients with a poor response to chemotherapy usually have an unfavorable prognosis [6]. The application of a risk-adapted strategy to evaluate the therapy response and prognosis ahead of therapy could improve personalized treatment. Therefore, identifying novel prognostic markers for improving the OS of osteosarcoma patients is particularly important.

Aging is characterized by gradual functional deterioration over time and results in increasing susceptibility to a variety of diseases, including cancers and cardiovascular, neurodegenerative, metabolic, and neoplastic diseases [7–10]. Cellular senescence is closely associated with aging [11, 12], and accumulating evidence suggested that senescence cells have a highly complex effect on tumors and can be both beneficial and detrimental. It can not only irreversibly arrest cell growth and inhibit cancer development [13–15] but also have the opposite effect and promote tumor malignancy via the secretion of senescence-associated secretory phenotype factors by the paracrine pathway [16, 17]. In the generation and regulation of cell aging, aging-related genes (ARGs) play a crucial role. It was confirmed that ARGs can not only inhibit tumors by regulating the cellular senescence of tumor cells but also potentially stimulate the initiation, metastasis, and development of tumors [13, 14, 18–20]. It has attracted great attention to identify key ARG characteristics and induce senescence of tumor cells [13]. The diagnostic or prognostic value of the ARG-based signature as biomarkers in malignancy has been widely studied [21, 22]. But the underlying mechanism and prognostic value of ARGs in osteosarcoma remain unclear, and a satisfactory ARG-based prognostic signature for osteosarcoma patients has not been reported.

In this study, by taking advantage of the TCGA database, we established molecular subtypes for osteosarcoma on the basis of prognosis-related ARGs and constructed an ARG-based risk signature for OS prediction of osteosarcoma patients. We compared the difference in clinicopathological features, immune infiltration, immune checkpoints, immunotherapy and chemotherapy response, and biological pathways based on molecular subtypes and the risk signature. The prognostic significance of the ARG-based prognostic signature was validated in GEO cohorts, and a nomogram composed of some clinicopathological factors and the risk signature was generated for providing an accurate prediction of osteosarcoma prognosis.

## 2. Materials and Methods

### 2.1. Data Collection and Preparation

Gene expression files and matched clinicopathological data of osteosarcoma patients were retrieved from TCGA and GEO. We removed cases without complete clinical information, follow-up of shorter than 30 days, or cases without status information. 85 patients from the TCGA cohort were retained as a training set, and 86 samples from the GSE21257 (53 cases) and GSE16091 (33 cases) cohorts were utilized for validation. The median value was taken as the gene expression value when a gene ID corresponded to multiple probes in the GEO cohorts or when multiple gene symbols existed in the TCGA cohort. From the Human Aging Genomic Resources (https://genomics.senescence.info/), a total of 307 human ARGs were obtained (Supplementary Table 1).

### 2.2. Consensus Clustering

The univariate Cox regression analysis filtered prognosis-correlated ARGs, and a heatmap was used to display the correlation among these prognosis-related ARGs. Consensus clustering analysis was conducted to generate ARG-related molecular subtypes using the “ConsensusClusterPlus” R package [23]. To compare the prognosis among the clusters, Kaplan-Meier (K-M) analysis was conducted.

### 2.3. Developing and Validating the Prognostic ARG Signature

The differentially expressed ARGs (deARGs) among the molecular subtypes were acquired by “limma” package under the screening conditions as the false discovery rate (FDR) < 0.05 and |log2 [fold change (FC)]| > log2(2) [24]. Then, prognosis-related ARGs were identified from the deARGs by univariate Cox regression analysis in the TCGA cohort. LASSO Cox regression analysis was applied to shrink the ARG number. Finally, to establish the prognostic signature, multivariate Cox regression analysis was then implemented. The formula for a risk signature was as follows:

(1) $$\begin{array}{c} \text{Risk Score}=\overset{n}{\underset{k=0}{\sum }}\beta_{i}\times {\text{Exp}}_{i}, \end{array}$$

where *β*_*i*_ represents the coefficient and Exp_*i*_ represents the normalized expression level of a gene. Osteosarcoma cases in the TCGA cohort and GEO cohort were divided into the high- and low-risk groups, according to the optimal cut-off point value of the risk score determined by maximally selected rank statistics using the “maxstat” R package (https://cran.r-project.org/web/packages/maxstat/index.html); here, a significant difference in prognosis between the two groups was detected. OS differences between the risk groups were compared by K-M analysis. The accuracy of the risk signature prediction was estimated by time-dependent ROC analysis in two sets (the validation and training sets).

### 2.4. Immune Infiltration, Chemotherapeutic Sensitivity, and Immunotherapy Response Predictions

The number of immune cells in the TCGA cohort was calculated using the xCell algorithm (https://xcell.ucsf.edu/) [25] that conducted cell type enrichment analysis for 64 immune cells based on gene expression data. The “estimate” R package was applied to estimate and extrapolate immune and stromal cell fraction in tumor samples [26]. In different groups, immune checkpoint expressions were compared. According to clusters and risks, to predict the clinical response to immune checkpoint inhibitors, the Tumor Immune Dysfunction and Exclusion (TIDE) (http://tide.dfci.harvard.edu/) algorithm was applied [27]. For predicting the chemosensitivity of osteosarcoma to several common anticancer drugs (methotrexate, paclitaxel, cisplatin, and doxorubicin), the “pRRophetic” R package was employed for determining half-maximal inhibitory concentration (IC_50_) values from osteosarcoma gene expression levels [28].

### 2.5. Gene Set Enrichment Analysis (GSEA)

To analyze the potential differences of pathways on the basis of molecular subtypes and ARG-related risk groups, GSEA was performed using hallmark gene sets as reference. The “GSVA” R package was used to carry out GSEA and pathways with the FDR < 0.05 being significantly enriched [29].

### 2.6. Establishment of a Predictive Nomogram

The decision tree model was applied to classify subgroups based on age, gender, metastatic status, and risk score by using the “rpart” R package (https://cran.r-project.org/web/packages/rpart/index.html). The independent prognostic factors of OS for osteosarcoma were identified by multivariate Cox regression analysis. By the “rms” R package (https://cran.r-project.org/web/packages/rms/index.html), we developed a nomogram integrating independent prognostic clinicopathological factors and the risk signature in the TCGA cohort. To evaluate the prediction accuracy between the actual observations and the predicted 1-, 3-, and 5-year OS probabilities, calibration curves were utilized. Time-dependent ROC curves assessed the nomogram discriminate ability. Decision curve analysis (DCA) tested the clinical applicability of the nomogram using “rmda” R package [30].

### 2.7. mRNA Expression-Based Stemness Indices (mRNAsi)

Based on the one-class logistic regression (OCLR) algorithm, the stemness index model trained from the Progenitor Cell Biology Consortium database was used to calculate tumor stemness. The stemness index can be used to measure how similar tumor cells are to stem cells, with the stemness index being a value between 0 (lowest) and 1 (highest). The closer the stemness index is to 1, the stronger the stem cell properties. We calculated transcriptome feature scores for the cohorts using the same Spearman correlation.

### 2.8. Statistical Analysis

The R software (v3.6.3) performed all of the statistical studies. The correlation matrices were conducted using the Pearson or Spearman correlation. The Wilcoxon test was conducted for two-group comparisons. Survival differences were compared using K-M curves with a log-rank test. A *P* value < 0.05 was considered statistically significant.

## 3. Results

### 3.1. Molecular Subtypes of ARG in Osteosarcoma

**Figure 1:**
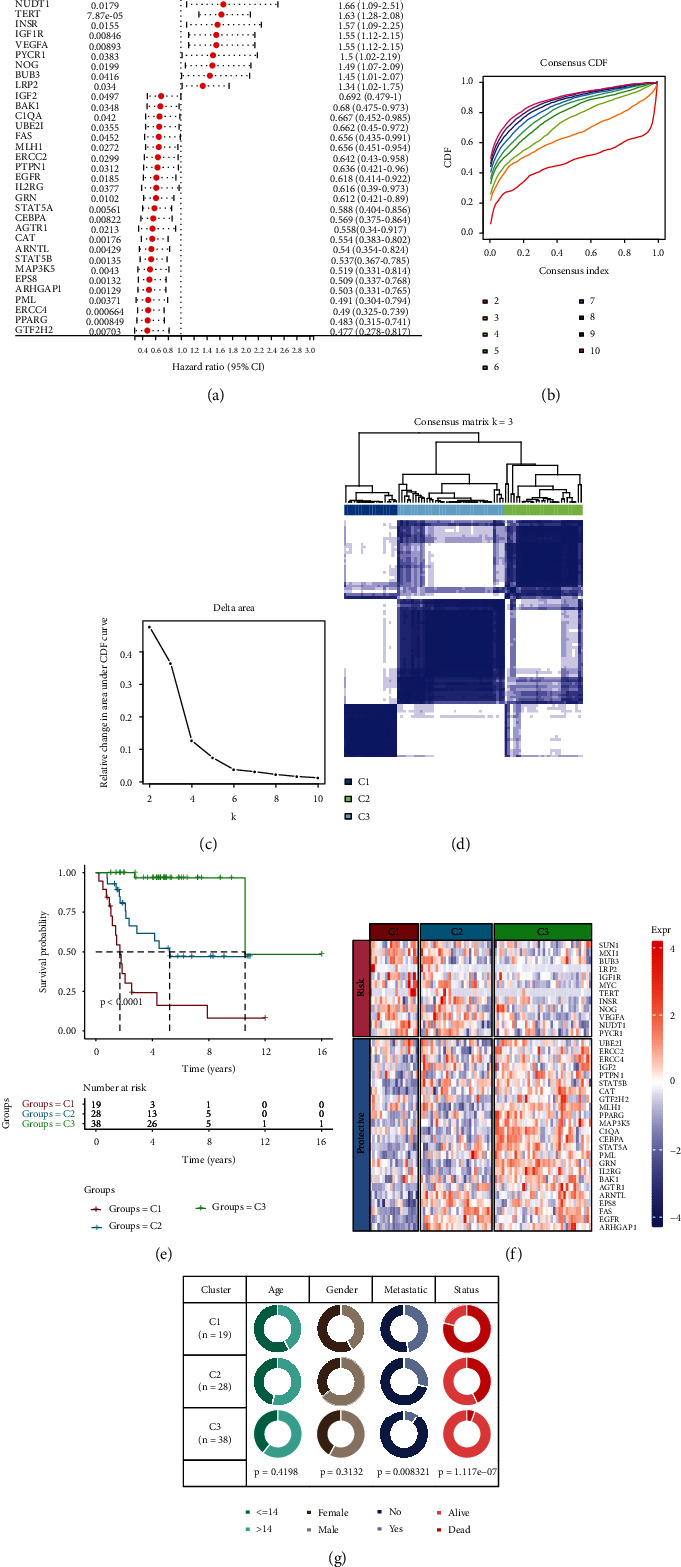
Construction of ARG-based patterns in osteosarcoma from the TCGA cohort. (a) The forest map showed multivariable Cox analysis of prognostic signatures. (b) Consensus cumulative distribution function (CDF) diagram with different *k* values. (c) Delta area plot for relative change in the area under CDF curve for *k* compared to *k* − 1. (d) Consensus matrix when number of groups (*k*) = 3. In the consensus matrix, white meant that samples were impossibly clustered together, and dark blue meant that samples were always clustered together. Both rows and columns of the matrix represented samples. (e) Kaplan-Meier curves for OS of three molecular subtypes. The survival probabilities were compared with log-rank test. (f) Clustering analysis of the expression of the 36 prognostic ARGs. (g) Comparison of clinicopathological characteristics among the C1, C2, and C3 clusters in the TCGA cohort.

We firstly identified 36 ARGs which were associated with osteosarcoma prognosis (Figure 1(a)). According to the expression profiles of the 36 ARGs, TCGA osteosarcoma patients were classified into C1, C2, and C3 with distinct outcomes (Figures 1(b)–1(d)). Those in the C3 subtype showed a longer survival than C1 and C2 subtypes (*P* < 0.0001, Figure 1(e)). Additionally, the expression level of the 36 prognosis-related ARGs is illustrated in Figure 1(f). Twelve genes belong to the “risk” group and are overall highly expressed in the C1 subtype, while the rest 24 genes were commonly upregulated in the C3 subtype and regarded as the “protective” genes. Moreover, as shown in Figure 1(g), osteosarcoma patients in the C1 subtype had higher tumor metastasis and mortality rate, followed by C2 and C3. Those data showed that 3 subtypes had clinical significance and may provide value for clinical diagnosis.

### 3.2. Differences in Immune Infiltration, Immunotherapy and Chemotherapy Response, and Biological Pathways among Molecular Subtypes

**Figure 2:**
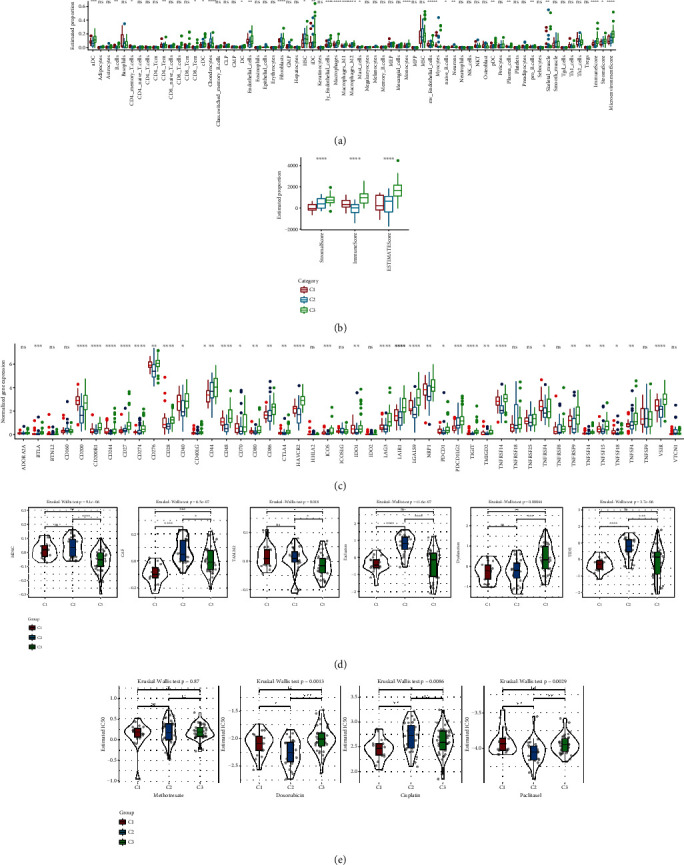
Association of tumor immune infiltration and response to immunotherapy and chemotherapy with the molecular subtypes in the TCGA cohort. (a) Differential expression analysis of immune cells. (b) Comparison of stromal score, immune scores, and ESTIMATE scores in patients with different molecular patterns. (c) Comparison of immune checkpoint-related genes in patients with different molecular patterns. (d) Comparison of MSDC score, CAF score, TAM.M2 score, T cell exclusion score, T cell dysfunction score, and TIDE score among C1, C2, and C3 subtypes. (e) The sensitivity of patients in different molecular patterns to methotrexate, cisplatin, cyclopamine, and paclitaxel. ns: no significance. ^∗^*P* < 0.05,  ^∗∗^*P* < 0.01,  ^∗∗∗^*P* < 0.001, and^∗∗∗∗^*P* < 0.0001.

The relative abundance of 64 immune cells in the TCGA cohort was assessed. Our data demonstrated that the estimated proportion of 40.625% (26/64) of immune cells were significantly different among the three subtypes (Figure 2(a)). The C3 subtype showed a significantly higher immune score than the C1 and C2 subtypes, indicating the highest immune infiltration of the C3 subtype (Figure 2(b)). Meanwhile, there were significant differences of the expression in the immune checkpoint-related gene among different subtypes, as shown in Figure 2(c). Subsequently, the TIDE algorithm was applied to assess the potential response to immunotherapy in the three molecular subtypes. As shown in Figure 2(d), we found that the C2 subtype has a higher TIDE score than C2 and C3 subtypes, indicating that osteosarcoma patients in the C1 and C3 clusters would be more reactive to immunotherapy compared with those in the C2 cluster. The C2 subtype was characterized by a significantly higher T cell exclusion and cancer-associated fibroblast (CAF) score than C1 and C3 subtypes, while the C3 subtype presented significantly higher myeloid-derived suppressor cells (MDSCs) and tumor-associated macrophages (TAMs), as well as a lower T cell dysfunction score. In addition, osteosarcoma patients in the C1 subtype showed a significantly lower IC_50_ to cisplatin than those in the C2 and C3 subtypes, whereas the C2 subtype showed a significantly lower IC_50_ to doxorubicin and paclitaxel than C1 and C3 subtypes, indicating that osteosarcoma patients in C1 were more sensitive to cisplatin, while patients in the C2 subtype were more sensitive to doxorubicin and paclitaxel (Figure 2(e)). Moreover, C1 had high mRNAsi (Supplementary Figure 1A).

### 3.3. Biological Pathways among Different Molecular Clusters

**Figure 3:**
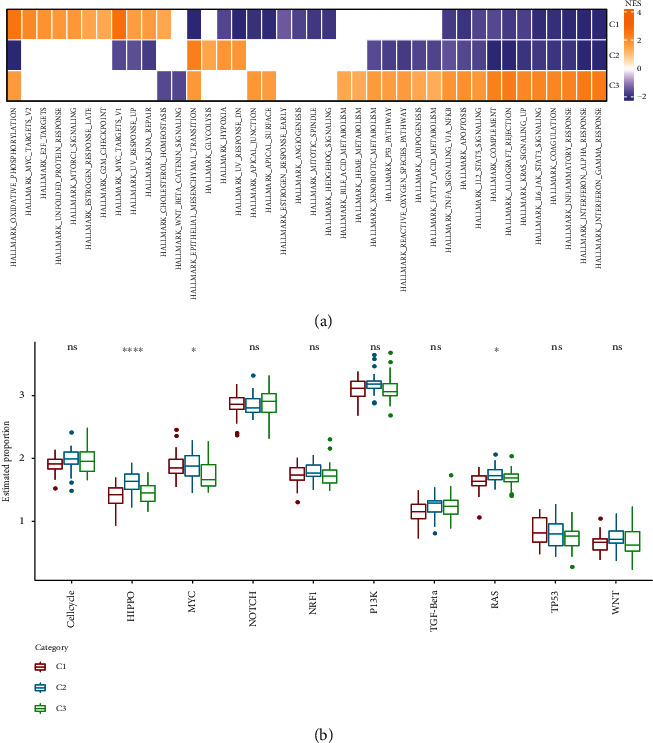
Gene Set Enrichment Analysis for osteosarcoma in the TCGA cohort. (a) Comparison of enriched pathways among the three molecular subtypes. (b) The estimated proportion of 10 oncogenic pathways among three molecular subtypes.

GSEA was performed to reveal the potential biological functions of the genes in the three molecular clusters. The results showed that the enriched pathways in these three subtypes are closely related to immunity. As shown in Figure 3(a), there are 31, 24, and 24 pathways enriched in the C1, C2, and C3 subtypes, respectively. In the C1 subtype, the enriched terms mainly consisted of suppressed immune-related pathways and activated cell cycle-related pathways. The immune-related pathways enriched in the C2 subtype were commonly suppressed, while the HALLMARK_EPITHELIAL_MESENCHYMAL_TRANSITION, HALLMARK_GLYCOLYSIS, HALLMARK_HYPOXIA, and HALLMARK_UV_RESPONSE_DN were activated. Most of the enriched immune-related pathways in the C3 subtype were activated. Additionally, the expression of HIPPO, MYC, and RAS was also significantly different among the three clusters (Figure 3(b)), which manifested as a higher expression level in the C2 subtype than the C1 and C3 subtypes.

### 3.4. Establishment and Evaluation of a Prognostic ARG-Based Risk Signature

**Figure 4:**
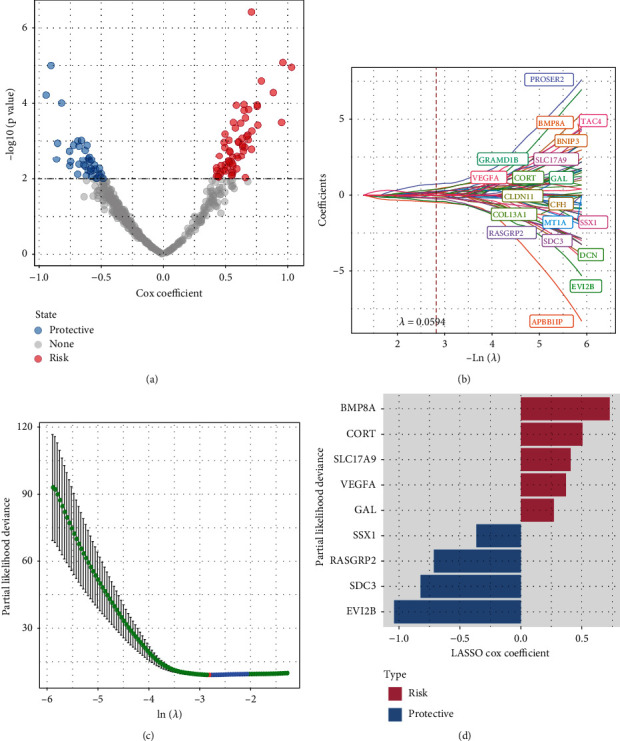
Construction of the ARG-based signature to predict prognosis of osteosarcoma in the TCGA cohort. (a) Volcano plot for ARGs in osteosarcoma. (b) The LASSO analysis was used to identify the prognostic variables and develop the predictive models. (c) Plots of the produced coefficient distributions for the logarithmic (lambda) series for parameter selection (lambda). (d) Multivariate Cox regression identification of an ARG-based risk signature for osteosarcoma prognosis prediction.

**Figure 5:**
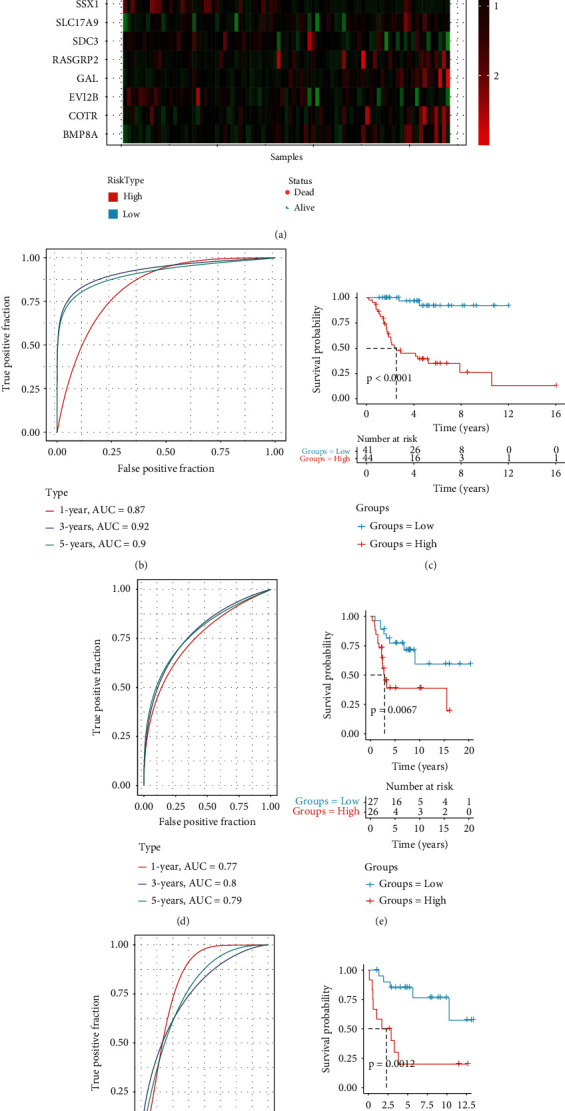
The ARG-based risk signature's prognostic importance in osteosarcoma. (a) The distribution of patient longevity status and risk score, and the expression profiles of nine aging genes in high- and low-risk groups in the TGGA cohort. (b, d, and f) The receiver operating characteristic curves for forecasting OS in TCGA, GSE21257, and GSE16091 cohorts, respectively. (c, e, and g) The survival curves for patients with high risk score and low risk score in the TCGA cohort.

495 differentially expressed ARGs were obtained by intersecting C1 vs. C2, C1 vs. C3, and C2 vs. C3, using the “limma” package. A total of 104 ARGs (*P* < 0.01) which contribute to osteosarcoma prognosis were screened from the 495 ARGs using univariate Cox regression analysis (Figure 4(a)). According to the results of the LASSO analysis, nineteen prognostic ARGs were further screened out based on the optimal lambda value (0.0594) (Figures 4(b) and 4(c)). Ultimately, we identified 9 ARGs in the risk score by multivariate Cox regression analysis (Figure 4(d)). Risk score = +0.506^∗^CORT − 0.714^∗^RASGRP2 − 0.822^∗^SDC3 + 0.728^∗^BMP8A + 0.271^∗^GAL + 0.408^∗^SLC17A9 − 1.04^∗^EVI2B + 0.37^∗^VEGFA − 0.365^∗^SSX1. Based on the risk score, osteosarcoma patients were successfully separated into the high- and low-risk groups (Figure 5(a)). Our results showed that patients with high risk exhibited shorter OS (*P* < 0.0001; Figure 5(c)) than those with low risk. The predictive efficacy of the risk signature was verified by the ROC curve (Figure 5(b)), with the AUC value for 1-, 3-, and 5-year OS being 0.87, 0.92, and 0.9, respectively. This was further validated in the GSE21257 and GSE16091 cohorts, as shown in Figures 5(d)–5(g). In addition, we determine the mRNAsi between the high group and the low group and found that there was no significance between the high group and the low group in the TCGA, GSE21257, and GSE16091 cohorts (Supplementary Figure 1B-D).

### 3.5. Correlation of Clinicopathological Characteristics with Risk Signature

**Figure 6:**
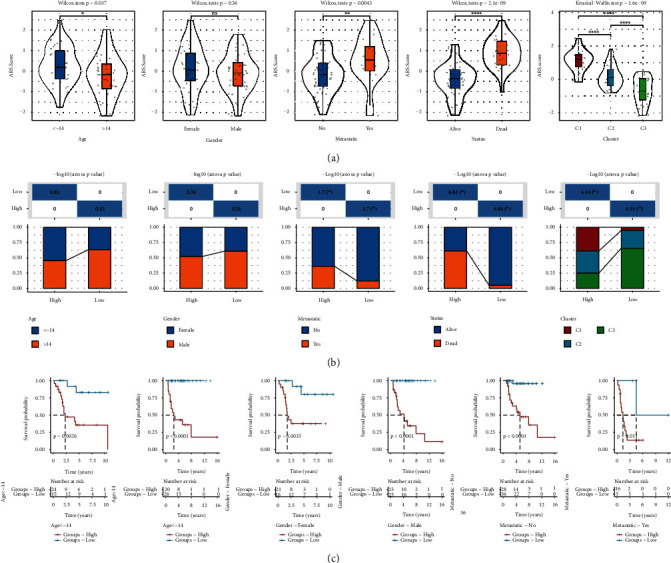
The correlations between the risk score and clinicopathological characteristics of osteosarcoma in the TCGA cohort. (a) The distribution of risk score in different groups separated by age, gender, metastasis, and status. (b) Comparison of age, gender, metastasis, status, and molecular subtypes between the high- and low-risk groups. (c) Kaplan-Meier survival subgroup analysis of all patients with osteosarcoma according to the risk signature stratified by clinical characteristics, including age, gender, and metastasis. The survival probabilities were compared with log-rank test. ns: no significance. ^∗^*P* < 0.05,  ^∗∗^*P* < 0.01,  ^∗∗∗^*P* < 0.001, and^∗∗∗∗^*P* < 0.0001.

We analyzed the relationship between clinicopathological features and the ARG-based risk model. The results showed that the risk score was significantly different in groups classified by age, metastasis, status, and molecular clusters (Figure 6(a)). Meanwhile, the high-risk group was characterized by a significantly higher metastasis, mortality rate, and proportion of C1 subtype than the low-risk group (Figure 6(b)). There was no significant difference between the high- and low-risk groups with regard to age and gender. Stratification analysis was conducted according to age, gender, and metastasis, and K-M survival analyses revealed that high-risk patients had significantly unfavorable survival outcomes compared with low-risk patients (Figure 6(c)). It suggested that the prognostic value of risk signature was applicable to other clinical features.

### 3.6. Immune Infiltration and Biological Pathways between Low- and High-Risk Groups

**Figure 7:**
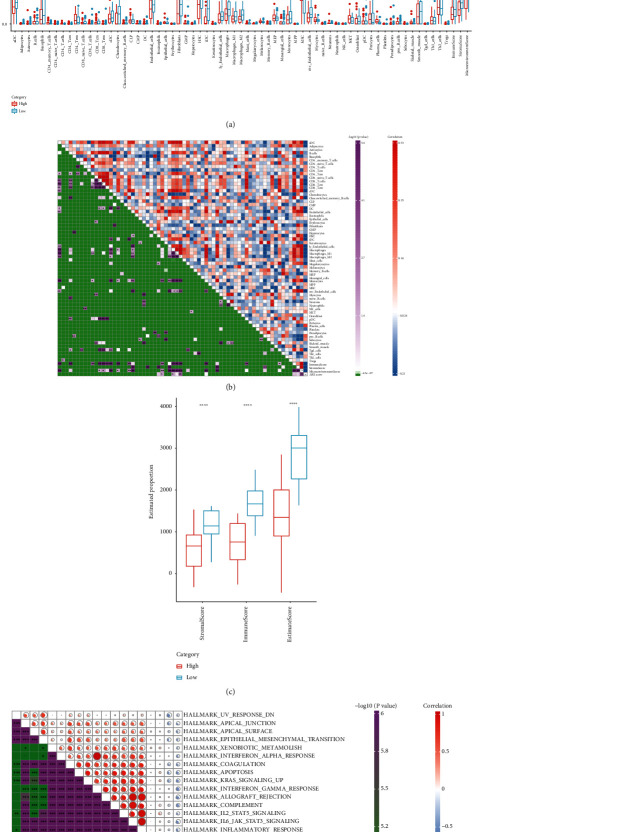
The characteristics of immune and pathway landscape between the high- and low-risk groups in the TCGA cohort. (a) Comparison of infiltration level of 64 immune cells in the high- and low-risk groups. (b) Correlation matrix of the risk score and infiltration level of immune cells. (c) Comparison of the stromal score, immune score, and ESTIMATE score between the high- and low-risk groups. (d) Correlation network between the enriched pathways and risk score. ns: no significance. ^∗^*P* < 0.05,  ^∗∗^*P* < 0.01,  ^∗∗∗^*P* < 0.001, and^∗∗∗∗^*P* < 0.0001.

As shown in Figure 7(a), eleven of the 64 tumor immune cell types showed significantly different abundance between the high- and low-risk groups. In the low-risk group, the multinucleated variant endothelial cells, macrophages M1, macrophages, lymphatic endothelial cells, lentivirus-induced dendritic cells (iDC), hematopoietic stem cells (HSC), fibroblasts, endothelial cells, and chondrocytes had higher infiltration levels than the high-risk group, while plasma cells and melanocytes in the high-risk group were significantly higher compared with the low-risk group in the TCGA cohort. The correlations between risk score and immune cell infiltration were heatmapped in Figure 7(b). It showed that the risk score was significantly negatively correlated with the infiltration level of M1 macrophages, lymphatic endothelial cells, and HSC. Additionally, compared with the high-risk group, patients in the low-risk group showed a higher stromal score and immune score, as well as a higher ESTIMATE score (Figure 7(c)). Biological pathways with a correlation greater than 0.3 are illustrated in Figure 7(d), and the majority of biological pathways were negatively correlated with the risk score. These findings indicated that the low group had higher immune infiltration.

### 3.7. Risk Signature Predicts Chemotherapy and Immunotherapy Response

**Figure 8:**
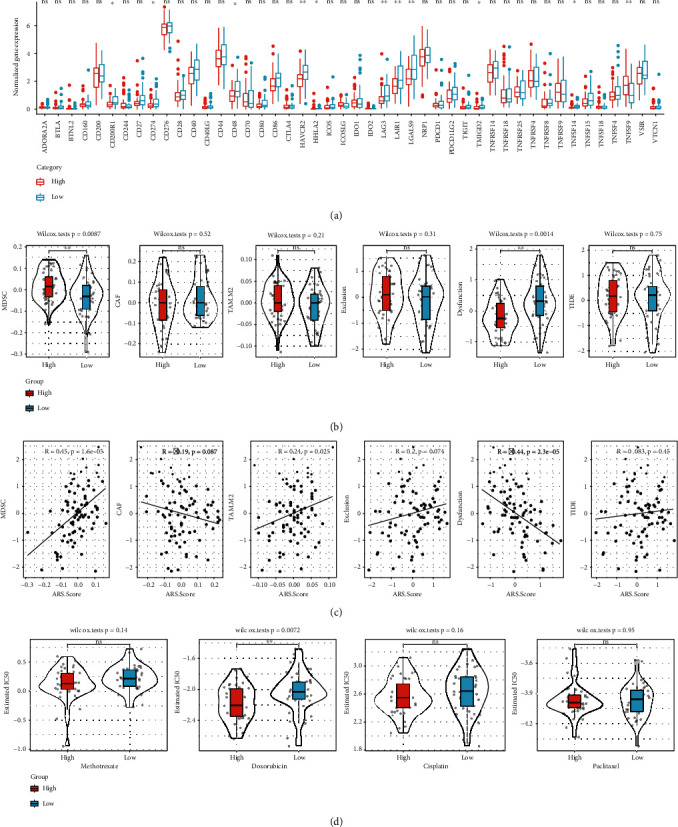
Comparison of response to immunotherapy and chemotherapy between the high- and low-risk groups in the TCGA cohort. (a) Comparison of gene expression of immune checkpoints. (b) Comparison of MSDC score, CAF score, TAM.M2 score, T cell exclusion score, T cell dysfunction score, and TIDE score. (c) Regression analysis of the TIDE results and the risk score. (d) The box plots of the estimated IC_50_ for cisplatin, doxorubicin, methotrexate, and paclitaxel. ns: no significance. ^∗^*P* < 0.05,  ^∗∗^*P* < 0.01,  ^∗∗∗^*P* < 0.001, and^∗∗∗∗^*P* < 0.0001.

We assessed the practicability of the risk signature in guiding systemic therapies in TCGA cohort. The expression of 11 immune checkpoints was significantly different between the high- and low-risk groups, including CD200R1, CD274, CD48, HAVCR2, HHLA2, LAG3, LAIR1, LGALS9, TMIGD2, TNFSF14, and TNFSF9 (Figure 8(a)). As shown in Figure 8(b), patients in the high-risk group had a higher MDSC score than those in the low-risk group. Nonetheless, there was no significant difference between the two risk groups with regard to the CAF score, TAM.M2 score, T cell exclusion score, T cell dysfunction score, and TIDE score. The risk score showed significant positive correlations with the MDSC score (*r* = 0.45, *P* = 1.6*e* − 5), TAM.M2 score (*r* = 0.24, *P* = 0.025), and T cell dysfunction score (*r* = 0.44, *P* = 2.3*e* − 5), as shown in Figure 8(c). We also found that the estimated IC_50_ values of doxorubicin were significantly higher in the high-risk group compared with that in the low-risk group (*P* = 0.0072). However, there was no significant difference between the risk score and the IC_50_ of methotrexate, paclitaxel, and cisplatin (Figure 8(d)). These findings indicated that a high risk score was related to elevated doxorubicin sensitivity.

### 3.8. Construction and Validation of a Nomogram

**Figure 9:**
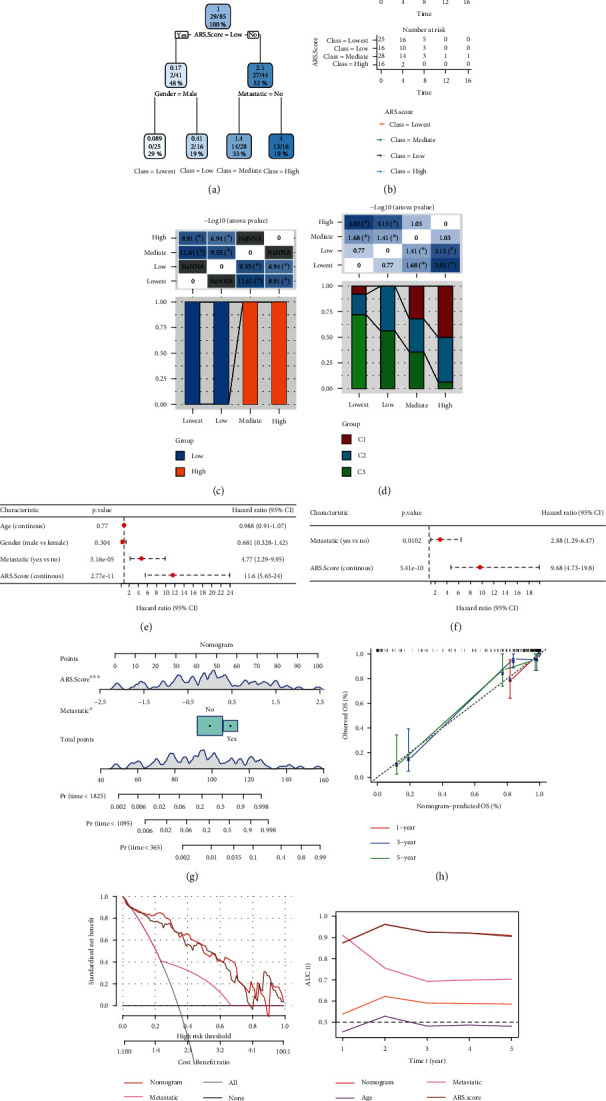
Construction and validation of a nomogram combining the risk signature and clinicopathological features. (a) Patients with full-scale annotations including risk score, metastatic, gender, and age were used to build a survival decision tree to optimize risk stratification. (b) Comparison of OS of the four subgroups obtained from the decision tree analysis in the TCGA cohort. (c) Correlations between the four subgroups and the risk signature. (d) Correlations between the four subgroups and molecular subtypes. (e) Univariate and (f) multivariate Cox analyses of risk score and clinicopathological characteristics in the TCGA cohort. (g) A nomogram combining risk signature and metastasis was generated in the TCGA cohort. (h) Comparison of the calibration curve for 1-, 3-, and 5-year OS of nomogram. (i) Decision curves for the clinical net benefit of each model in comparison to all or none strategies. The *x*-axis indicated the threshold probability, and the *y*-axis indicated the net clinical benefit. (j) Time-dependent ROC curves comparing the prognostic accuracy of nomogram, age, gender, metastasis, and risk score in the TCGA cohort.

We constructed a decision tree based on age, gender, metastasis, and risk score of osteosarcoma patients in the TCGA cohort (Figure 9(a)), and the results showed that the patients can be stratified into four distinct groups (lowest, low, mediate, and high) using a decision tree on only risk score, gender, and metastatic status. K-M survival analysis showed that there was a significant difference in OS among the four groups (Figure 9(b)). Patients in the “lowest” and “low” subgroups belong to the ARG-based low-risk group (Figure 9(c)). In addition, C1 and C2 subtypes occupy more than the C3 subtype in the “highest” group (Figure 9(d)). Multivariate Cox regression analysis demonstrated that the risk score was the most significant independent prognostic factor of osteosarcoma (HR = 9.68, 95% CI: 4.73-19.80, *P* = 5.41*e* − 10), followed by metastatic status (HR = 2.88, 95% CI: 1.29-6.47, *P* = 0.0102) (Figures 9(e) and 9(f)). Therefore, a nomogram is then generated using the risk score and metastatic status to predict the OS of osteosarcoma patients (Figure 9(g)). The calibration plot demonstrated that the nomogram can effectively forecast the actual survival outcomes (Figure 9(h)). Moreover, the DCA curve and time-ROC analysis demonstrated that the nomogram and risk signature had better prognostic capacity than other clinicopathological features, as shown in Figures 9(i) and 9(j).

## 4. Discussion

Osteosarcoma is the most common bone sarcoma with high heterogeneity and has various subtypes based on morphological and molecular characteristics [31]. Herein, we established three ARG-based patterns with distinct diversity for osteosarcoma patients. The cellular constitutions of stromal and immune cells in the tumor microenvironment (TME) are involved in osteosarcoma progression, chemotherapy resistance, and immunosuppressive activities [32]. In this study, the C3 subtype had a distinctly higher stromal and immune score than C1 and C2, implying the potential differences among the clusters with regard to the progression and response to chemotherapy and immunotherapy. Previous evidence suggested that patients with higher stromal or immune scores had a favorable OS of osteosarcoma [33]. Consistently, the C3 subtype exhibited prolonged survival time and lower mortality and metastasis rates. Although advances in chemotherapy improved the prognosis of osteosarcoma patients [34], drug resistance would result in worse clinical outcomes [35]. Here, patients in the C2 cluster showed more responsiveness to paclitaxel and doxorubicin, while osteosarcoma patients in the C1 subtype were higher reactive to cisplatin. These data suggested that these patients were more likely to benefit from these chemotherapy drugs.

T cell immune checkpoint molecules serve as promising immunotherapeutic targets for tumor, and their inhibitors have dramatically changed the therapeutic landscape of osteosarcoma patients with metastasis or recurrence [36]. Nevertheless, few parts of osteosarcoma patients could benefit from immunotherapies [37]. C1 and C3 subtypes presented increased infiltration levels of many immune cells and higher TIDE scores. Meanwhile, our GSEA results revealed that immune-related pathways were significantly inhibited in the C1 subtype and activated in the C3 subtype. These findings suggested that patients in C1 and C2 subtypes were more likely to respond to immunotherapies. Furthermore, our data found significant differences among the molecular subtypes with regard to M2-TAMs, MDSCs, CAFs, and cytotoxic T lymphocytes (CTLs), which contribute to tumor growth, invasion, and metastasis and mediate the communication between malignant cells, immune cells, and stromal cells [38].

With the development of omic technology and public databases, a variety of risk signatures for prognosis prediction in osteosarcoma has been established on the basis of clinicopathological factors and omic characteristics. Accumulating evidence has elucidated the potential capability of radiomics [39], DNA methylation [40], and immune-related genes [41] for predicting the prognosis of osteosarcoma. Recently, ARG-based prognostic models for various cancers have been established and displayed satisfactory performance for prognosis prediction [42, 43]. At present, the prognostic role of the ARGs in osteosarcoma is still unclear, and an ARG-based risk model has not been developed. Here, we constructed and verified an ARG-based risk signature, which possessed a good performance in the prediction of clinical outcomes of osteosarcoma patients. ARGs can not only inhibit tumor growth but also promote tumor invasion and metastasis [13]. In our ARG-based risk model, osteosarcoma patients with a high risk score were characterized by higher metastasis and poorer prognosis, indicating that ARGs in osteosarcoma were closely associated with cancer progression. Further, we constructed a predictive nomogram integrating the risk score and metastatic status for osteosarcoma, which presented good calibration and discrimination and had adequate ability to predict survival outcomes in patients with osteosarcoma.

Accumulating studies have reported the associations between the ARGs in our risk signature and osteosarcoma. Bone morphogenetic protein 8a (BMP8A), a number of the bone morphogenetic protein ligand, encodes a secreted ligand of the TGF-*β* superfamily of proteins. It has been proved to promote survival and drug resistance in clear cell renal cell carcinoma (ccRCC) [44], but studies about its effect on osteosarcoma are rare. Cortistatin (CORT) encodes a neuropeptide that is structurally similar to somatostatin. A recent report showed that CORT could inhibit the proliferation of the human thyroid carcinoma cell line, indicating a possible inhibitory role of CORT in cancer development [45]. Solute carrier family 17 member 9 (SLC17A9) was involved in the progression of colorectal cancer (CRC) and breast cancer and was recognized as a potential biomarker for outcome prediction of CRC and BC patients [46, 47]. Vascular endothelial growth factor A (VEGFA) is a potent angiogenic factor for blood vessel formation, and research has proved that VEGFA participates in the angiogenesis and progression of osteosarcoma [48]. Galectin-1 (GAL-1) functions in tissue development, cell proliferation, and immunoregulation. The expression of galectin-1 was capable to be used to differentiate small-cell osteosarcoma from Ewing sarcoma [49]. SSX family member 1 (SSX1) has been associated with stem cell migration, suggesting a potential biologically important role in the metastatic phenotype [50]. RAS guanyl releasing protein 2 (RASGRP2) encodes a brain-enriched nucleotide exchanged factor, and research demonstrated that abnormal expression of RASGRP2 in lung adenocarcinoma correlated with the infiltration level of immune cells [51]. Syndecan 3 (SDC3) encodes a protein that belongs to the syndecan proteoglycan family. Upregulation of SDC3 contributes to perineural invasion and poor outcomes in pancreatic cancer [52]. A recent study has confirmed that the ecotropic viral integration site 2B (EVI2B) could be used as a new prognostic biomarker for metastatic melanoma [53]. However, the functions and underlying mechanisms of these ARGs have not been exhaustively investigated in osteosarcoma.

Cellular senescence and immune infiltration in the TME were proved to contribute to the response of immunotherapy [54], but the correlations between immune infiltration and senescence in osteosarcoma remain poorly understood. Our data revealed that osteosarcoma patients with low risk scores showed an apparent increase in immune cell infiltration, which manifests as a significantly elevated infiltration of multinucleated variant endothelial cells, macrophages M1, macrophages, lymphatic endothelial cells, iDC, HSC, fibroblasts, endothelial cells, and chondrocytes. Moreover, we found that the risk score was negatively correlated with the immune-related pathways. A higher expression level of ten immune checkpoints was observed in the low-risk group, indicating that the patients with lower risk scores had a higher potential to benefit from immunotherapy. However, osteosarcoma patients in the low-risk group had a lower TNFSF9 expression, which was associated with immune response and cell growth in osteosarcoma [55]. Furthermore, evidence suggested that chemosensitivity was regulated by ARGs in osteosarcoma patients [56]. Our data revealed that osteosarcoma patients in the high-risk group were more reactive to doxorubicin. A recent study showed that BMP8A can diminish chemotherapy sensitivity in ccRCC by promoting Nrf2 phosphorylation and activating TRIM24 [44], implying its potential role in drug resistance in osteosarcoma.

Nevertheless, several limitations in our study should be acknowledged. First, the molecular subtypes and ARG-based risk signature were generated using retrospective data from public databases. Therefore, it should be validated in more prospective and multicenter osteosarcoma cohorts in the future. Second, we only investigated the potential prognostic value of the ARG risk signature, so further studies are required to explore the underlying mechanisms of the signature in the development of osteosarcoma.

## 5. Conclusions

In summary, this study identified molecular subtypes based on ARGs and developed an ARG-based survival prognostic model for osteosarcoma. The difference in immune landscape, biological functions, drug sensitivity, and immunotherapy response according to molecular subtypes and risk groups were analyzed. A nomogram combining the novel ARGs-based risk model and metastatic status was constructed. It may serve as a clinical tool for making personalized therapeutic treatments and forecasting prognoses for osteosarcoma patients.

## Data Availability

The datasets analyzed in this study could be found in GSE21257 at https://www.ncbi.nlm.nih.gov/geo/query/acc.cgi?acc=GSE21257 and in GSE16091 at https://www.ncbi.nlm.nih.gov/geo/query/acc.cgi?acc=GSE21257.

## Supplementary Materials

Supplementary 1 Supplementary Table 1: the list of 307 ARGs downloaded from the Human Aging Genomic Resources 3.

Supplementary 2 Supplementary Figure 1: mRNAsi analysis: (A) the mRNAsi analysis in 3 clusters; (B) the mRNAsi analysis between high group and low group in the TCGA dataset; (C) the mRNAsi analysis between high group and low group in the GSE21257 dataset; and (D) the mRNAsi analysis between high group and low group in the GSE16091 dataset.
