# Supplementary material for: Establishing and Validating an Aging-Related Prognostic Signature in Osteosarcoma
Source: Stem Cells Int. 2023 Feb 23;2023:6245160. doi: 10.1155/2023/6245160 (PMC10643040; doi:10.1155/2023/6245160)
Supplement: Supplementary 1 — Supplementary Table 1: the list of 307 ARGs downloaded from the Human Aging Genomic Resources 3. [file 6245160.f1.docx]

**Supplementary Tables 1:** The list of 307 ARGs downloaded from the Human Aging Genomic Resources 3.

| **Symbol** | **Gene name** |
| --- | --- |
| GHR | growth hormone receptor |
| GHRH | growth hormone releasing hormone |
| SHC1 | SHC (Src homology 2 domain containing) transforming protein 1 |
| POU1F1 | POU class 1 homeobox 1 |
| PROP1 | PROP paired-like homeobox 1 |
| TP53 | tumor protein p53 |
| TERC | telomerase RNA component |
| TERT | telomerase reverse transcriptase |
| ATM | ATM serine/threonine kinase |
| PLAU | plasminogen activator, urokinase |
| ERCC2 | excision repair cross-complementation group 2 |
| ERCC8 | excision repair cross-complementation group 8 |
| WRN | Werner syndrome, RecQ helicase-like |
| LMNA | lamin A/C |
| IGF1R | insulin-like growth factor 1 receptor |
| TXN | thioredoxin |
| KL | klotho |
| E2F1 | E2F transcription factor 1 |
| PTPN11 | protein tyrosine phosphatase, non-receptor type 11 |
| NFKB2 | nuclear factor of kappa light polypeptide gene enhancer in B-cells 2 (p49/p100) |
| STAT5B | signal transducer and activator of transcription 5B |
| STAT3 | signal transducer and activator of transcription 3 (acute-phase response factor) |
| STAT5A | signal transducer and activator of transcription 5A |
| NRG1 | neuregulin 1 |
| HDAC3 | histone deacetylase 3 |
| GH1 | growth hormone 1 |
| IL7R | interleukin 7 receptor |
| IGF1 | insulin-like growth factor 1 (somatomedin C) |
| IGF2 | insulin-like growth factor 2 |
| INS | insulin |
| NGF | nerve growth factor (beta polypeptide) |
| IRS1 | insulin receptor substrate 1 |
| PTPN1 | protein tyrosine phosphatase, non-receptor type 1 |
| IRS2 | insulin receptor substrate 2 |
| AKT1 | v-akt murine thymoma viral oncogene homolog 1 |
| PIK3CB | phosphatidylinositol-4,5-bisphosphate 3-kinase, catalytic subunit beta |
| NGFR | nerve growth factor receptor |
| HRAS | Harvey rat sarcoma viral oncogene homolog |
| MYC | v-myc avian myelocytomatosis viral oncogene homolog |
| EGFR | epidermal growth factor receptor |
| ERBB2 | erb-b2 receptor tyrosine kinase 2 |
| INSR | insulin receptor |
| NCOR1 | nuclear receptor corepressor 1 |
| NBN | nibrin |
| JUND | jun D proto-oncogene |
| IL2 | interleukin 2 |
| PDGFB | platelet-derived growth factor beta polypeptide |
| EGF | epidermal growth factor |
| IL2RG | interleukin 2 receptor, gamma |
| FOS | FBJ murine osteosarcoma viral oncogene homolog |
| PDGFRB | platelet-derived growth factor receptor, beta polypeptide |
| EPOR | Erythropoietin receptor |
| SST | somatostatin |
| PRKCD | protein kinase C, delta |
| PPARA | peroxisome proliferator-activated receptor alpha |
| RET | ret proto-oncogene |
| PLCG2 | phospholipase C, gamma 2 (phosphatidylinositol-specific) |
| PEX5 | peroxisomal biogenesis factor 5 |
| TCF3 | transcription factor 3 |
| PARP1 | poly (ADP-ribose) polymerase 1 |
| BRCA1 | breast cancer 1, early onset |
| PIN1 | peptidylprolyl cis/trans isomerase, NIMA-interacting 1 |
| PTEN | phosphatase and tensin homolog |
| CREBBP | CREB binding protein |
| HIF1A | hypoxia inducible factor 1, alpha subunit (basic helix-loop-helix transcription factor) |
| UBB | ubiquitin B |
| RPA1 | replication protein A1, 70kDa |
| BLM | Bloom syndrome, RecQ helicase-like |
| BCL2 | B-cell CLL/lymphoma 2 |
| S100B | S100 calcium binding protein B |
| VCP | valosin containing protein |
| POLG | polymerase (DNA directed), gamma |
| IGFBP3 | insulin-like growth factor binding protein 3 |
| HSP90AA1 | heat shock protein 90kDa alpha (cytosolic), class A member 1 |
| NR3C1 | nuclear receptor subfamily 3, group C, member 1 (glucocorticoid receptor) |
| EGR1 | early growth response 1 |
| VEGFA | vascular endothelial growth factor A |
| ABL1 | ABL proto-oncogene 1, non-receptor tyrosine kinase |
| BRCA2 | breast cancer 2, early onset |
| TOP2A | topoisomerase (DNA) II alpha |
| TOP2B | topoisomerase (DNA) II beta |
| NFKB1 | nuclear factor of kappa light polypeptide gene enhancer in B-cells 1 |
| TOP1 | topoisomerase (DNA) I |
| RAD51 | RAD51 recombinase |
| UBE2I | ubiquitin-conjugating enzyme E2I |
| TNF | tumor necrosis factor |
| PDPK1 | 3-phosphoinositide dependent protein kinase 1 |
| CEBPA | CCAAT/enhancer binding protein (C/EBP), alpha |
| CEBPB | CCAAT/enhancer binding protein (C/EBP), beta |
| MXI1 | MAX interactor 1, dimerization protein |
| TGFB1 | transforming growth factor, beta 1 |
| ERCC6 | excision repair cross-complementation group 6 |
| STK11 | serine/threonine kinase 11 |
| EP300 | E1A binding protein p300 |
| APTX | aprataxin |
| PML | promyelocytic leukemia |
| GSK3B | glycogen synthase kinase 3 beta |
| HTT | huntingtin |
| PRKCA | protein kinase C, alpha |
| SSTR3 | somatostatin receptor 3 |
| HELLS | helicase, lymphoid-specific |
| APOC3 | apolipoprotein C-III |
| EEF2 | eukaryotic translation elongation factor 2 |
| ERCC3 | excision repair cross-complementation group 3 |
| TERF1 | telomeric repeat binding factor (NIMA-interacting) 1 |
| PRKDC | protein kinase, DNA-activated, catalytic polypeptide |
| CAT | catalase |
| ERCC5 | excision repair cross-complementation group 5 |
| AR | androgen receptor |
| GTF2H2 | general transcription factor IIH, polypeptide 2, 44kDa |
| XRCC5 | X-ray repair complementing defective repair in Chinese hamster cells 5 (double-strand-break rejoining) |
| PCNA | proliferating cell nuclear antigen |
| FEN1 | flap structure-specific endonuclease 1 |
| FAS | Fas cell surface death receptor |
| TERF2 | telomeric repeat binding factor 2 |
| XRCC6 | X-ray repair complementing defective repair in Chinese hamster cells 6 |
| POLD1 | polymerase (DNA directed), delta 1, catalytic subunit |
| BAX | BCL2-associated X protein |
| RB1 | retinoblastoma 1 |
| EMD | emerin |
| GRB2 | growth factor receptor-bound protein 2 |
| FOXO3 | forkhead box O3 |
| FOXO1 | forkhead box O1 |
| HSF1 | heat shock transcription factor 1 |
| XPA | xeroderma pigmentosum, complementation group A |
| MSRA | methionine sulfoxide reductase A |
| RECQL4 | RecQ helicase-like 4 |
| SOD2 | superoxide dismutase 2, mitochondrial |
| SOD1 | superoxide dismutase 1, soluble |
| FOXM1 | forkhead box M1 |
| COQ7 | coenzyme Q7 homolog, ubiquinone (yeast) |
| CACNA1A | calcium channel, voltage-dependent, P/Q type, alpha 1A subunit |
| LRP2 | low density lipoprotein receptor-related protein 2 |
| AIFM1 | apoptosis-inducing factor, mitochondrion-associated, 1 |
| UCHL1 | ubiquitin carboxyl-terminal esterase L1 (ubiquitin thiolesterase) |
| APP | amyloid beta (A4) precursor protein |
| APOE | apolipoprotein E |
| A2M | alpha-2-macroglobulin |
| SNCG | synuclein, gamma (breast cancer-specific protein 1) |
| PRDX1 | peroxiredoxin 1 |
| PON1 | paraoxonase 1 |
| RELA | v-rel avian reticuloendotheliosis viral oncogene homolog A |
| IL6 | interleukin 6 |
| RGN | regucalcin |
| ATP5O | ATP synthase, H+ transporting, mitochondrial F1 complex, O subunit |
| RAD52 | RAD52 homolog, DNA repair protein |
| TOP3B | topoisomerase (DNA) III beta |
| ERCC1 | excision repair cross-complementation group 1 |
| SIRT1 | sirtuin 1 |
| HDAC1 | histone deacetylase 1 |
| HSPA9 | heat shock 70kDa protein 9 (mortalin) |
| GPX1 | glutathione peroxidase 1 |
| GSR | glutathione reductase |
| GSS | glutathione synthetase |
| GSTA4 | glutathione S-transferase alpha 4 |
| GSTP1 | glutathione S-transferase pi 1 |
| MT-CO1 | mitochondrially encoded cytochrome c oxidase I |
| HSPD1 | heat shock 60kDa protein 1 (chaperonin) |
| HSPA1A | heat shock 70kDa protein 1A |
| HSPA1B | heat shock 70kDa protein 1B |
| PCMT1 | protein-L-isoaspartate (D-aspartate) O-methyltransferase |
| MAPK8 | mitogen-activated protein kinase 8 |
| YWHAZ | tyrosine 3-monooxygenase/tryptophan 5-monooxygenase activation protein, zeta |
| PTK2B | protein tyrosine kinase 2 beta |
| PTK2 | protein tyrosine kinase 2 |
| IL7 | interleukin 7 |
| MAPK14 | mitogen-activated protein kinase 14 |
| FGFR1 | fibroblast growth factor receptor 1 |
| SP1 | Sp1 transcription factor |
| FLT1 | fms-related tyrosine kinase 1 |
| JUN | jun proto-oncogene |
| MED1 | mediator complex subunit 1 |
| MAPK9 | mitogen-activated protein kinase 9 |
| MAPK3 | mitogen-activated protein kinase 3 |
| HMGB1 | high mobility group box 1 |
| CCNA2 | cyclin A2 |
| HMGB2 | high mobility group box 2 |
| MAP3K5 | mitogen-activated protein kinase kinase kinase 5 |
| TAF1 | TAF1 RNA polymerase II, TATA box binding protein (TBP)-associated factor, 250kDa |
| LMNB1 | lamin B1 |
| SDHC | succinate dehydrogenase complex, subunit C, integral membrane protein, 15kDa |
| FOXO4 | forkhead box O4 |
| HESX1 | HESX homeobox 1 |
| PIK3R1 | phosphoinositide-3-kinase, regulatory subunit 1 (alpha) |
| BSCL2 | Berardinelli-Seip congenital lipodystrophy 2 (seipin) |
| AGPAT2 | 1-acylglycerol-3-phosphate O-acyltransferase 2 |
| BMI1 | BMI1 proto-oncogene, polycomb ring finger |
| EEF1A1 | eukaryotic translation elongation factor 1 alpha 1 |
| TFAP2A | transcription factor AP-2 alpha (activating enhancer binding protein 2 alpha) |
| BDNF | brain-derived neurotrophic factor |
| CREB1 | cAMP responsive element binding protein 1 |
| ATF2 | activating transcription factor 2 |
| TBP | TATA box binding protein |
| APEX1 | APEX nuclease (multifunctional DNA repair enzyme) 1 |
| HBP1 | HMG-box transcription factor 1 |
| BUB1B | BUB1 mitotic checkpoint serine/threonine kinase B |
| PTGS2 | prostaglandin-endoperoxide synthase 2 (prostaglandin G/H synthase and cyclooxygenase) |
| HSPA8 | heat shock 70kDa protein 8 |
| SIN3A | SIN3 transcription regulator family member A |
| CDK1 | cyclin-dependent kinase 1 |
| TFDP1 | transcription factor Dp-1 |
| DDIT3 | DNA-damage-inducible transcript 3 |
| POLA1 | polymerase (DNA directed), alpha 1, catalytic subunit |
| MAPT | microtubule-associated protein tau |
| CTGF | connective tissue growth factor |
| HDAC2 | histone deacetylase 2 |
| MAX | MYC associated factor X |
| MXD1 | MAX dimerization protein 1 |
| MDM2 | MDM2 proto-oncogene, E3 ubiquitin protein ligase |
| SUMO1 | small ubiquitin-like modifier 1 |
| H2AFX | H2A histone family, member X |
| HOXB7 | homeobox B7 |
| HOXC4 | homeobox C4 |
| JAK2 | Janus kinase 2 |
| ESR1 | estrogen receptor 1 |
| LEP | leptin |
| LEPR | leptin receptor |
| NFKBIA | nuclear factor of kappa light polypeptide gene enhancer in B-cells inhibitor, alpha |
| CLU | clusterin |
| MTOR | mechanistic target of rapamycin (serine/threonine kinase) |
| GHRHR | growth hormone releasing hormone receptor |
| CTNNB1 | catenin (cadherin-associated protein), beta 1, 88kDa |
| PSEN1 | presenilin 1 |
| DLL3 | delta-like 3 (Drosophila) |
| CDKN2A | cyclin-dependent kinase inhibitor 2A |
| PPP1CA | protein phosphatase 1, catalytic subunit, alpha isozyme |
| DBN1 | drebrin 1 |
| NOG | noggin |
| ELN | elastin |
| ATR | ATR serine/threonine kinase |
| UCP3 | uncoupling protein 3 (mitochondrial, proton carrier) |
| ZMPSTE24 | zinc metallopeptidase STE24 |
| TP63 | tumor protein p63 |
| UCP2 | uncoupling protein 2 (mitochondrial, proton carrier) |
| POLB | polymerase (DNA directed), beta |
| GCLC | glutamate-cysteine ligase, catalytic subunit |
| GCLM | glutamate-cysteine ligase, modifier subunit |
| SIRT6 | sirtuin 6 |
| BUB3 | BUB3 mitotic checkpoint protein |
| RAE1 | ribonucleic acid export 1 |
| PMCH | pro-melanin-concentrating hormone |
| MLH1 | mutL homolog 1 |
| CSNK1E | casein kinase 1, epsilon |
| STUB1 | STIP1 homology and U-box containing protein 1, E3 ubiquitin protein ligase |
| PPM1D | protein phosphatase, Mg2+/Mn2+ dependent, 1D |
| CHEK2 | checkpoint kinase 2 |
| PCK1 | phosphoenolpyruvate carboxykinase 1 (soluble) |
| ARHGAP1 | Rho GTPase activating protein 1 |
| CDC42 | cell division cycle 42 |
| ARNTL | aryl hydrocarbon receptor nuclear translocator-like |
| CLOCK | clock circadian regulator |
| HIC1 | hypermethylated in cancer 1 |
| PAPPA | pregnancy-associated plasma protein A, pappalysin 1 |
| ADCY5 | adenylate cyclase 5 |
| PPARGC1A | peroxisome proliferator-activated receptor gamma, coactivator 1 alpha |
| GPX4 | glutathione peroxidase 4 |
| UCP1 | uncoupling protein 1 (mitochondrial, proton carrier) |
| FGF23 | fibroblast growth factor 23 |
| EFEMP1 | EGF containing fibulin-like extracellular matrix protein 1 |
| ERCC4 | excision repair cross-complementation group 4 |
| CETP | cholesteryl ester transfer protein, plasma |
| PPARG | peroxisome proliferator-activated receptor gamma |
| AGTR1 | angiotensin II receptor, type 1 |
| CISD2 | CDGSH iron sulfur domain 2 |
| EEF1E1 | eukaryotic translation elongation factor 1 epsilon 1 |
| EPS8 | epidermal growth factor receptor pathway substrate 8 |
| KCNA3 | potassium channel, voltage gated shaker related subfamily A, member 3 |
| SIRT7 | sirtuin 7 |
| SLC13A1 | solute carrier family 13 (sodium/sulfate symporter), member 1 |
| SOCS2 | suppressor of cytokine signaling 2 |
| TPP2 | tripeptidyl peptidase II |
| TP53BP1 | tumor protein p53 binding protein 1 |
| SIRT3 | sirtuin 3 |
| NCOR2 | nuclear receptor corepressor 2 |
| SUN1 | Sad1 and UNC84 domain containing 1 |
| BAK1 | BCL2-antagonist/killer 1 |
| IGFBP2 | insulin-like growth factor binding protein 2, 36kDa |
| PYCR1 | pyrroline-5-carboxylate reductase 1 |
| TP73 | tumor protein p73 |
| CNR1 | cannabinoid receptor 1 (brain) |
| NFE2L2 | nuclear factor, erythroid 2-like 2 |
| CDKN1A | cyclin-dependent kinase inhibitor 1A (p21, Cip1) |
| PDGFRA | platelet-derived growth factor receptor, alpha polypeptide |
| PIK3CA | phosphatidylinositol-4,5-bisphosphate 3-kinase, catalytic subunit alpha |
| C1QA | complement component 1, q subcomponent, A chain |
| CDKN2B | cyclin-dependent kinase inhibitor 2B (p15, inhibits CDK4) |
| EIF5A2 | eukaryotic translation initiation factor 5A2 |
| MIF | macrophage migration inhibitory factor (glycosylation-inhibiting factor) |
| DGAT1 | diacylglycerol O-acyltransferase 1 |
| MT1E | metallothionein 1E |
| FGF21 | fibroblast growth factor 21 |
| HTRA2 | HtrA serine peptidase 2 |
| GSK3A | glycogen synthase kinase 3 alpha |
| NUDT1 | nudix (nucleoside diphosphate linked moiety X)-type motif 1 |
| IKBKB | inhibitor of kappa light polypeptide gene enhancer in B-cells, kinase beta |
| SQSTM1 | sequestosome 1 |
| CDK7 | cyclin-dependent kinase 7 |
| GRN | granulin |
| SERPINE1 | serpin peptidase inhibitor, clade E (nexin, plasminogen activator inhibitor type 1), member 1 |
| SPRTN | SprT-like N-terminal domain |
| RICTOR | RPTOR independent companion of MTOR, complex 2 |
| CTF1 | cardiotrophin 1 |
| TRAP1 | TNF receptor-associated protein 1 |
| TRPV1 | transient receptor potential cation channel subfamily V member 1 |
| NFE2L1 | nuclear factor, erythroid 2-like 1 |
| IFNB1 | Interferon beta |
| GDF11 | growth differentiation factor 11 |
