# Supplementary figures and images for: Establishing and Validating an Aging-Related Prognostic Signature in Osteosarcoma
Source: Stem Cells Int. 2023 Feb 23;2023:6245160. doi: 10.1155/2023/6245160 (PMC10643040; doi:10.1155/2023/6245160)

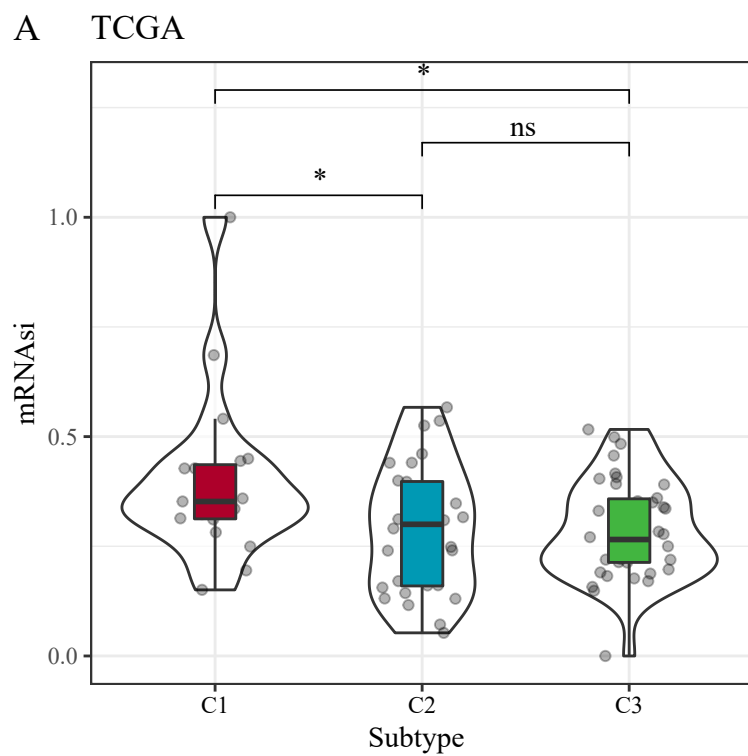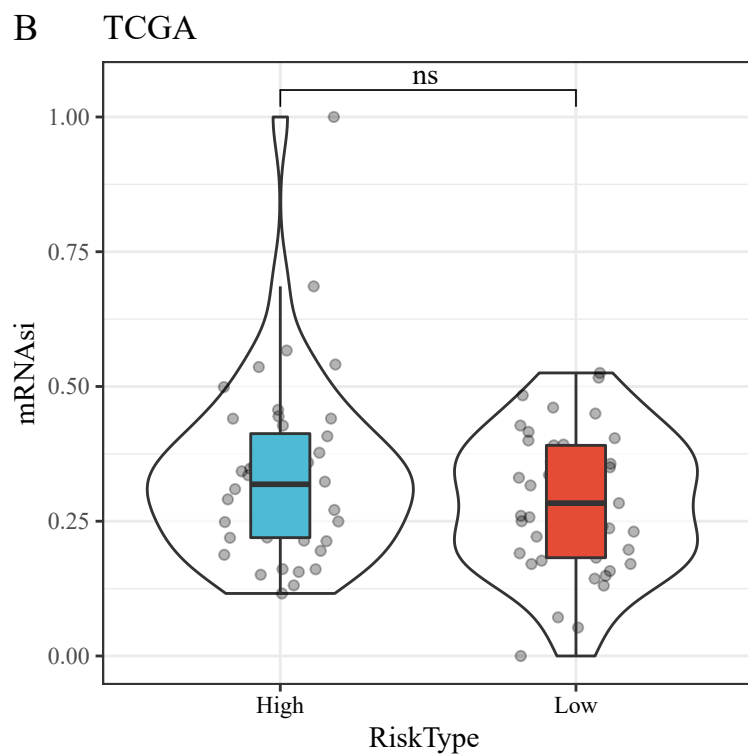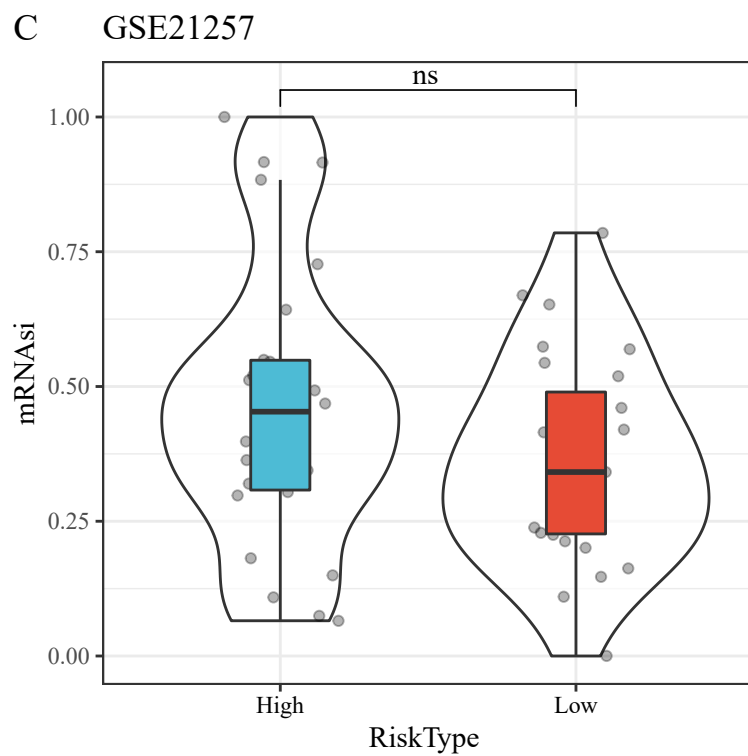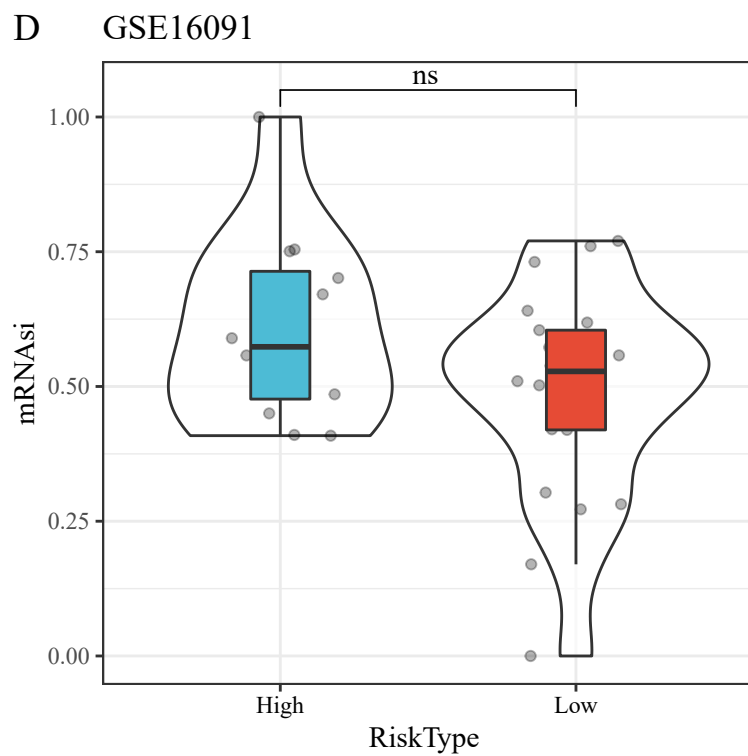

Supplement: Supplementary 2 — Supplementary Figure 1: mRNAsi analysis: (A) the mRNAsi analysis in 3 clusters; (B) the mRNAsi analysis between high group and low group in the TCGA dataset; (C) the mRNAsi analysis between high group and low group in the GSE21257 dataset; and (D) the mRNAsi analysis between high group and low group in the GSE16091 dataset. [file 6245160.f2.pdf]
